# Dynamic Mechanical Response and Damage Mechanism of HTPB Propellant under Impact Loading

**DOI:** 10.3390/ma13133031

**Published:** 2020-07-07

**Authors:** Hengning Zhang, Meng Liu, Yinggang Miao, Han Wang, Tao Chen, Xuezhong Fan, Hai Chang

**Affiliations:** 1 Xi’an Modern Chemistry Research Institute, Xi’an 710049, Shaanxi, China; zhn3216@163.com (H.Z.); mcri204lm@163.com (M.L.); mcri204wh@163.com (H.W.); mcri204ct@163.com (T.C.); mcri204fxz@163.com (X.F.); 2 Department of Engineering Mechanics, School of Aerospace Engineering, State Key Laboratory for Strength and Vibration of Mechanical Structures, Xi’an Jiaotong University, Xi’an 710049, Shaanxi, China; miaoyinggang@xjtu.edu.cn

**Keywords:** hydroxyl-terminated polybutadiene propellant, split Hopkinson pressure bar, strain rate sensitivity, damage mechanisms

## Abstract

The dynamic mechanical behaviors of Hydroxyl-terminated polybutadiene (HTPB) propellant was studied by a split Hopkinson pressure bar apparatus (SHPB) at strain rates ranging from 10^3^ to 10^4^ s^−1^. The obtained stress–strain curves indicated that the mechanical features, such as ultimate stress and strain energy, were strongly dependent on the strain rate. The real time deformation and fracture evolution of HTPB propellant were captured by a high-speed digital camera accompanied with an SHPB setup. Furthermore, microscopic observation for the post-test specimen was conducted to explore the different damage mechanisms under various conditions of impact loading. The dominated damage characteristics of HTPB propellant were changed from debonding and matrix tearing to multiple cracking modes of ammonium perchlorate (AP) particles, along with the increase of the strain rate. For the first time, the influence of AP particle density on the dynamic response of HTPB propellant was studied by analyzing the strain-rate sensitivity (SRS) index of HTPB propellant with two different filler content (80 wt.% and 85 wt.%), which deduced from a power function of ultimate stress and strain energy density. The result of this study is of significance for evaluating the structural integrity and security of HTPB propellant.

## 1. Introduction

As an important type of solid propellant, Hydroxyl-terminated polybutadiene (HTPB) propellant has been universally equipped in solid rocket motors (SRMs) for both military and civil use, because of its outstanding advantages, such as high energy level, good processability and excellent mechanical properties [1,2,3]. In terms of material composition, HTPB propellant is a typical highly filled heterogeneous polymer composite, including ammonium perchlorate (AP) and aluminum particles as solid fillers, HTPB as a binder and other additives [4].

HTPB propellant, served as energy sources in a missile, is more vulnerable than the other components of a missile system under severe impulsive loadings [5,6,7,8,9,10,11]. Specifically, the solid propellant grain is usually exposed to different forms of impact loadings, such as accidental dropping, launch overload and fragment impact under attack, which correspond with strain rates spanning from 10^3^ s^−1^ to 10^4^ s^−1^ [12]. These types of impact loading can cause severe failure and even lead to unwilling initiation of the solid propellant, which constitutes a great menace for the survivability and reliability of missile weapons [13,14,15,16]. Therefore, it is of great importance to study the characteristics of the mechanical performance and failure mode of HTPB propellant under impact loadings. Moreover, these studies can provide the supporting data for numerical simulations, which is beneficial to assess the safety and structural integrity of HTPB propellant.

Recently, numerous studies have focused on the quasi-static and dynamic behaviors of solid propellants at different strain rates. For example, Wang et al. conducted a series of compressive tests of HTPB propellant over a wide range of strain rates (from 1.7 × 10^−4^ to 2500 s^−1^) [17]. They believed that the stress is linearly related to the logarithm of strain rate when the strain rate ranged from 1.7 × 10^−4^ to 1 s^−1^, whereas the relation turns to act as an exponential function while the strain rate ranged from 1 to 2500 s^−1^. Wang et al. also examined the rate dependence on the yield stress of composite modified double base (CMDB) propellant with a similar method. They indicated that the yield stress increases bilinearly with the logarithm of strain rate ranging from 1.7 × 10^−4^ to 4000 s^−1^. The transition of rate dependence on both HTPB propellant and CMDB propellant can be ascribed to the different molecular motion unit under low and high strain rate loading [18]. Sun et al. investigated the compressive behaviors of a CMDB propellant at strain rates ranging from 10^−4^ s^−1^ to 10^3^ s^−1^ [19]. The curves of stress–strain showed linear viscoelastic, yielding and strain hardening or strain softening successively. Chen et al. studied the dynamic compressive damage mechanisms of HTPB propellant at strain rates ranging from 700 to 1900 s^−1^, using a split Hopkinson pressure bar apparatus (SHPB) apparatus [20]. The scanning electron microscopy (SEM) observation exhibited three damage modes of HTPB propellant under high-speed compressive loading, transgranular fracture, increasing porosity and matrix tearing. Ho et al. examined the damage mechanisms of three typical propellants that imposed a high strain rate loading (ca.1000 s^−1^), involving a “brittle-ductile” procedure when increasing the temperature [21]. Drodge et al. quantified the impact damage of the RDX-HTPB composites at a strain rate of 1000 s^−1^, by measuring four damage metrics, the compressive strength, the modulus, the thermal conductivity and the porosity [22]. Zhang et al. obtained the high strain rate (up to 4500 s^−1^) compressive stress–strain curves for a novel NEPE propellant over a temperature range from −40 °C to +40 °C, using a modified SHPB device [23]. Sunny conducted a modified SHPB testing on an HTPB polymer and its composite for comparison [24], the strain rate dependence indicated a remarkable transition at 2100 s^−1^.

Although there are studies on the dynamic compression tests of HTPB propellant that refer to strain rates ranging from 10^−4^ to 10^3^ s^−1^, information on the impact response and damage mechanisms of HTPB propellant at strain rates in the order of 10^4^ s^−1^ is still limited and for which strain rates correspond to dynamic circumstances of high-speed impact, such as accidental dropping, launch overload and fragment impact. Furthermore, HTPB propellant is a typical highly filled heterogeneous composite, and the filler particle density has an appreciable effect on the dynamic mechanical properties and damage characteristics of HTPB propellant. However, the influence of particle density on the dynamic compressive response of HTPB propellant is largely unexplored, according to the existing literature.

In this work, an SHPB apparatus was applied to obtain the dynamic compressive response of HTPB propellant with strain rates spanning from 780 to 8960 s^−1^ at room temperature. Another goal of this work is to introduce an empirical formula to quantitatively analyze the strain-rate sensitivity index of ultimate stress and strain energy density of HTPB propellant by fitting the mechanical data. SEM images of the post-test specimen were conducted to explore the different damage characteristics after dynamic loading. Furthermore, the mechanical properties of HTPB propellants with two different filler contents (80 wt.% and 85 wt.%) were compared to analyze the influence of AP particle density on high-speed impact response of HTPB propellant for the first time.

## 2. Experimental Methodology

### 2.1. Material and Specimen Preparation

The investigated HTPB propellant specimen was fabricated by a typical slurry casting method, including with HTPB as polymer binder, AP and aluminum particles as solid fillers, DOA as a plasticizer and other additives (including Ferrocene derivatives and lead based oxides as combustion catalyst, stannous octanoate as curing catalyst, 2, 4-Toluene diisocyanate as curing agent and so on). Herein, the number average molecular weight of HTPB binder is approximately 3500–4800, the hydroxyl value and average hydroxyl functionality of HTPB is 0.50–0.80 mmol/g and 2.18, respectively. The AP particles are irregular crystals with sizes ranging from 80 to 150 μm, while aluminum particles are spherical-shaped grains with a size distribution from 12 to 18 μm in a normal form, which can be attributed to the preparation process, and 15 μm is the mean size. Generally, HTPB propellants embedding 80 to 85 wt.% of filler density are more widely accepted for the well-balanced, good performance of fabrication and high energy level. The constitution of two HTPB propellants with 80 and 85 wt.% filler content is listed in Table 1, which is denoted as H-80W and H-85W, respectively.

**Figure 1:**
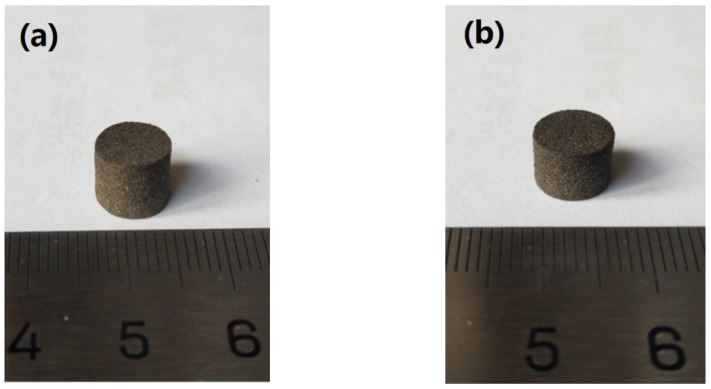
Photographs of two sizes of machined specimens for the (**a**) low-strain-rate loading test and (**b**) high-strain-rate loading test.

Components of HTPB propellant were blended in a kneading machine for 2 h in a slurry form, then the slurry was poured into the prepared molds. After being cured in an oven at temperatures of 50 °C for 6 days, the obtained HTPB propellant was machined into cylinders with two dimensions, as shown in Figure 1. The specimen in Figure 1a has a geometry size of 9 mm diameter and 9 mm length, which is used for low-strain-rate loading (780 to 1980 s^−1^ in this study). The other has a geometry size of 9 mm diameter and 6 mm length, which is used for high-strain-rate loading (2450 to 8960 s^−1^ in this study), because a higher length-to-diameter ratio is beneficial for the reduction of stress wave attenuation in the high strain-rate loading [25]. After machining, both two sizes of samples were kept in an oven at 50 °C for more than 24 h to eliminate the residual stress.

### 2.2. Dynamic Compressive Mechanical Testing

**Figure 2:**
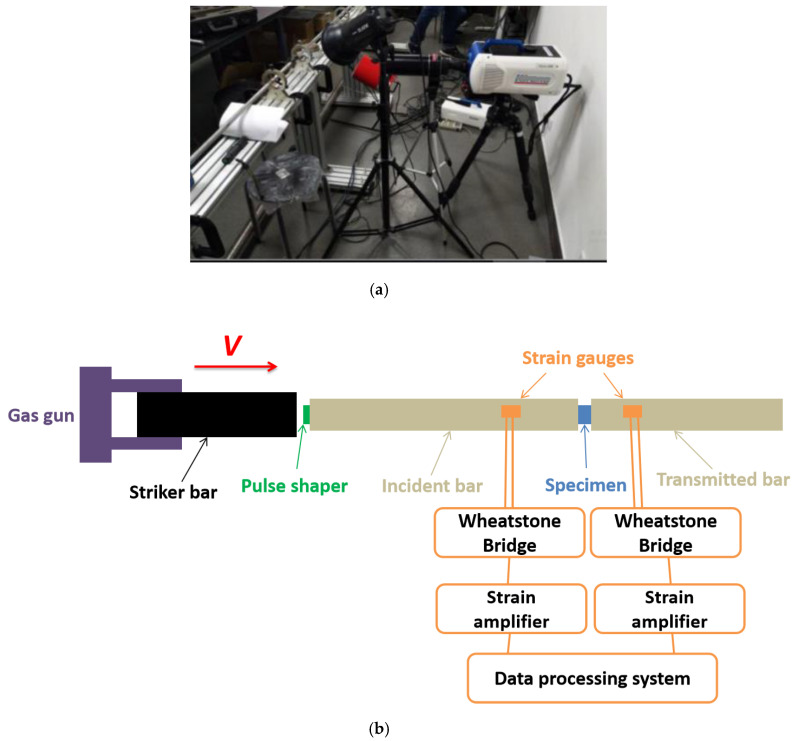
(**a**) A photograph of the split Hopkinson pressure bar device fitted with a high-speed digital camera. (**b**) Scheme of the split Hopkinson pressure bar device.

Since Kolsky developed the SHPB technology for the dynamic mechanical test [26], the SHPB tests have been extensively applied to study the mechanical properties of various materials under strain rates ranging from 10^2^ to 10^5^ s^−1^, such as metals, rocks, composite materials and solid propellant [27,28,29,30,31,32,33,34,35,36]. In this paper, an SHPB device was equipped to perform the impact loading experiment on the HTPB propellant. The photograph of the SHPB apparatus fitted with a high-speed digital camera was depicted in Figure 2a, and the schematic of the SHPB device is depicted in Figure 2b.

During the test, the sample was located between the transmitted bar and the incident bar. Initially, the compressed air pulsed the striker bar, which was fired by a gas gun, then the striker bar impacted the incident bar with a specific velocity, V, generating a compressive stress pulse on one end face of the incident bar, then the stress pulse propagated through the incident bar. The strain gauges recorded the stress pulse, which was mounted on the incident bar. When the stress wave arrived at the boundary between the sample and the incident bar, part of the pulse traveled through the transmitted bar, while the remaining part was reflected back to the incident bar. All types of strain signals were recorded and magnified by the strain gauges and strain amplifier, respectively. Thanks to the pulse shaper technology, which can minimize the oscillations and achieve a dynamic stress equilibrium. A film of lubricant between the bars and sample was utilized to reduce the interfacial friction. Based on the strain signals recorded by strain gauges, the relationship with the strain rate, strain, and stress of sample, can be expressed by using Equation (1) [37,38,39,40].

(1) $$\left\{ \begin{array}{l} {\dot{\varepsilon }}_{engi.}=-\frac{2C_{B}}{l_{s}}\varepsilon_{r}\\ \varepsilon_{engi.}=-\frac{C_{B}}{l_{s}}\int_{0}^{t}\varepsilon_{r}dt\\ \sigma_{engi.}=E_{B}\cdot \frac{A_{B}}{A_{s}}\varepsilon_{t} \end{array}\right.$$

where *C_B_*, *A_B_*, and *E_B_* are the wave velocity, cross sectional area and Young’s modulus of the bars, respectively. The wave velocity of the bars can be expressed as, *C_B_* =$\;\sqrt{E/\rho }$, in which *ρ* is the bar’s mass density. *l_s_* and *A_s_* are the length and cross sectional area of the sample before the test. *ε_r_* and *ε_t_* are the recorded strain signals of the incident bar and transmitted bar, respectively. Based on the hypothesis of a constant volume of sample during the whole deformation, the true strain *ε_true_* and true stress *σ_true_* can be associated with engineering strain *ε_engi_* and engineering stress *σ_engi_* by using Equation (2) [41,42].

(2) $$\left\{ \begin{array}{l} \varepsilon_{true}=\ln(1+\varepsilon_{engi.})\\ \sigma_{true}=\sigma_{engi.}(1+\varepsilon_{engi.}) \end{array}\right.$$

**Figure 3:**
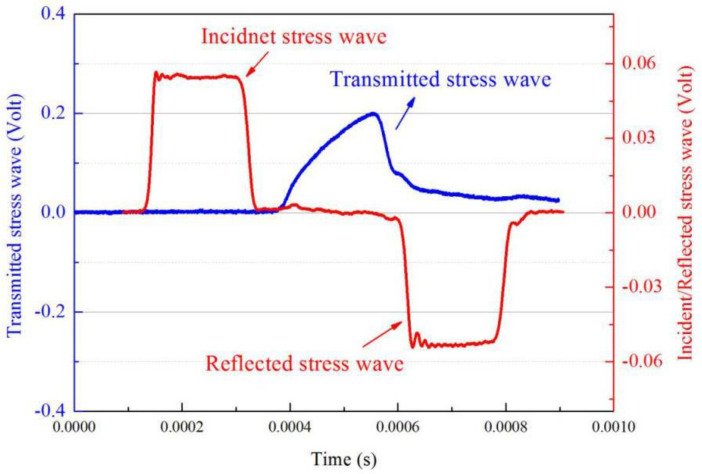
Original signals of incident, reflected, and transmitted waves recorded by the strain gauges.

Figure 3 illustrates the original signals of incident, reflected, and transmitted waves in an SHPB test on H-80W at a strain rate of 2460 s^−1^. The pulse duration can be determined as 200 μs from Figure 3. Thanks to the filtration of pulse shaper, the high frequency components can be distinctly identified from the incident signal, which is essential to minimize the experimental error. The rectangular shaped region of the reflected signal corresponds to a constant amplitude, indicating that the sample experienced a constant engineering strain rate deformation under dynamic stress equilibrium. For the SHPB tests in this study, at least three samples were tested by repeating each strain rate of loading, to ensure that the obtained stress–strain curves were reliable. Meanwhile, the real time procedure of deformation in an SHPB test is synchronously recorded by a high-speed digital camera. A post-test SEM was applied to investigate the microscopic features of impact-induced damage.

## 3. Results and Discussion

### 3.1. Dynamic Stress–Strain Characteristics and Fracture Behavior

**Figure 4:**
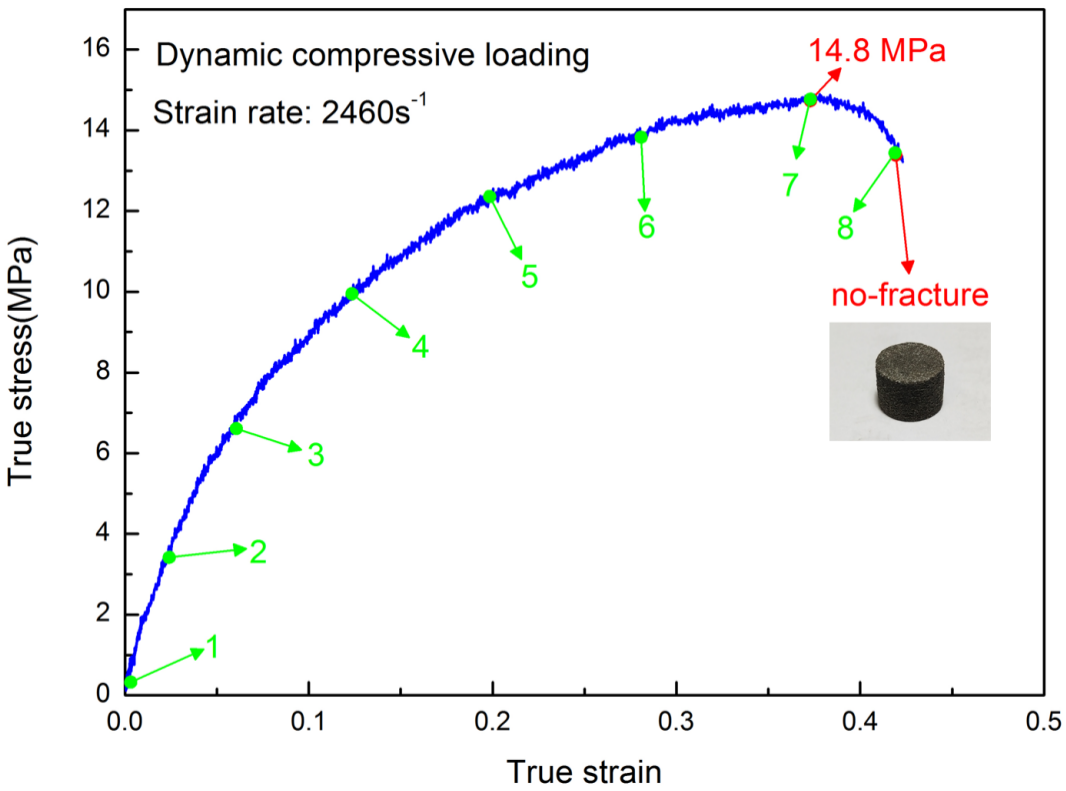
True stress–strain curve of H-80W under the strain rate of 2460 s^−1^ without causing an ultimate fracture. The green numbers in the curve correspond to the numbers marked in the images recorded by a high-speed digital camera in Figure 5.

Herein, H-80W was chosen as the representative for dynamic experiments at various strain rates. H-85W was introduced to study the influence of AP particle density on the dependence of strain rate, by comparing with H-80W. Figure 4 shows the true stress–strain curve of H-80W under a strain rate of 2460 s^−1^. From Figure 4, a nonlinear elastic deformation behavior is displayed, without an obvious yielding, the true stress keeps rising to a peak value of 14.8 MPa (ultimate stress) at a strain of 0.42 and a time of about 200 μs, the time of which is equal to the pulse duration for the current SHPB test.

**Figure 5:**
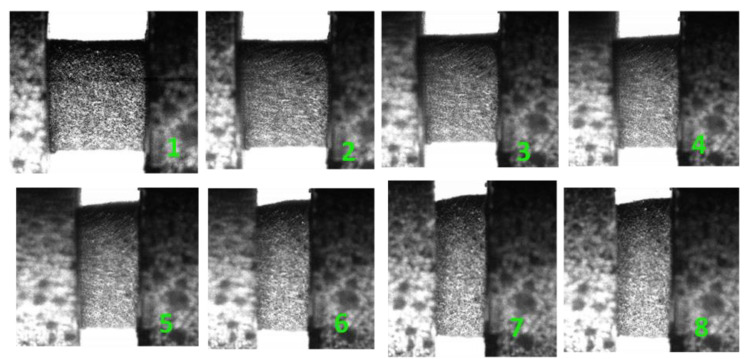
Deformation processes of H-80W recorded by a high-speed digital camera under a strain rate of 2460 s^−1^. The green numbers in the images correspond to the numbers marked in Figure 4.

In this SHPB test, the strain energy density represented by the area below the stress–strain curve can be determined as 4.75 MJ/m^3^, which indicates the total energy consumption of H-80W at a strain rate of 2460 s^−1^. The real time deformation photographs of H-80W were recorded by a high-speed digital camera, and the green numbers in the stress–strain curve correspond to the numbers marked in the high-speed photographs in Figure 5. As shown in image No. 1 in Figure 5, a cylinder-shaped sample was initially located in the middle of two bars. From images No. 2 to No. 4 image in Figure 5, a generally homogeneous deformation of H-80W occurs, which corresponds to a stress equilibrium in the sample during test. The status of homogeneous deformation continues until a strain of 0.15, of which the stress is 10 MPa. From image No. 5 (strain of 0.2 and stress of 12 MPa), a barreling behavior takes place. It means that the one-dimensional deformation of the specimen tends to be disturbed by the interfacial friction effect. However, for this test, the influence of interfacial friction on the stress state can be minimized, because the grease was used as lubricant and the inhomogeneous deformation of the specimen is not pronounced. At the final part of the stress–strain curve, the unloading procedure initiates before the specimen was imposed to totally fracture, indicating that the strain energy density of 4.75 MJ/m^3^ is insufficient to destroy H-80W.

**Figure 6:**
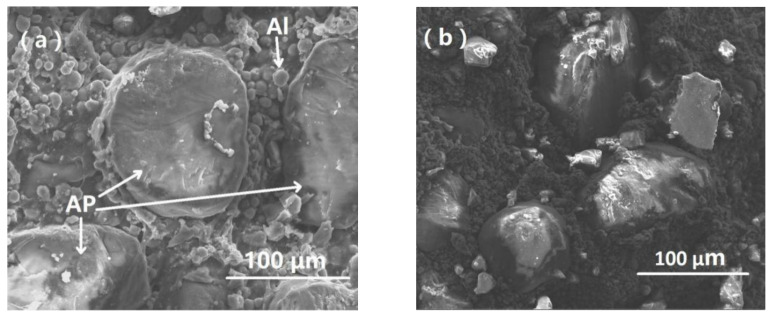

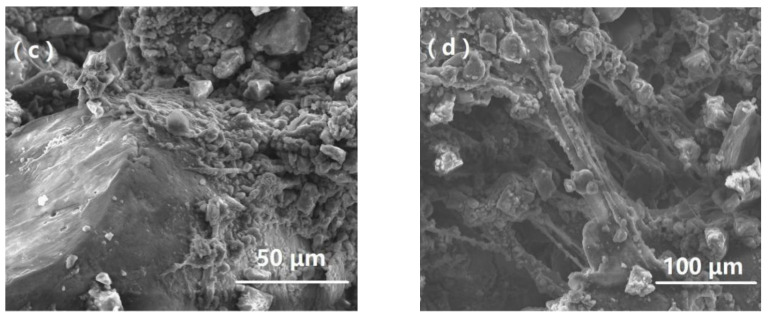
SEM micrographs of the non-fractured H-80W after split Hopkinson pressure bar apparatus (SHPB) testing at room temperature under a strain rate of 2460 s^−1^: (**a**) ammonium perchlorate (AP) particles embedding within the HTPB matrices as well as the distinct interfaces of the particle-binder in an untested specimen; (**b**) debonding of the particle–binder interface induced by impact loading; (**c**) the unbroken AP particle with covering residual binder polymer; (**d**) the strip-shape polymer caused by severe binder matrix tearing.

**Figure 7:**
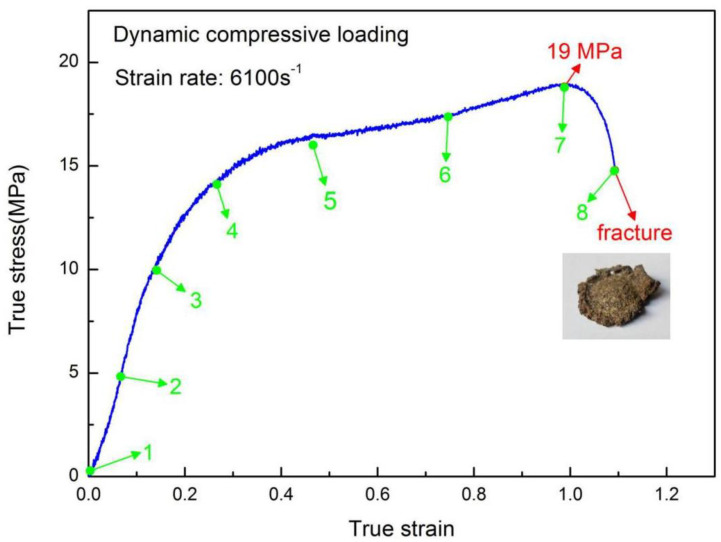
True stress-strain curve of H-80W under a strain rate of 6100 s^−1^, causing a final fracture, the green numbers in the curve correspond to the numbers marked in the images recorded by a high-speed digital camera in Figure 8.

As Figure 5 depicted, no macroscopic damage or cracking was observed under a strain rate of 2460 s^−1^. In order to examine the damage characteristics of H-80W at a microscopic level, SEM observation was employed to study the impact face of the post-test sample, and the direction of impact loading is perpendicular to the photograph. From Figure 6a, AP particles were uniformly dispersed in the binder matrix and the AP particle-binder interfaces can be clearly observed in an untested sample. As well, the distribution of spherical aluminum particles also can be found with a dimension in the order of several microns. The debonding behavior of the particle-binder interface induced by impact loading can be revealed in Figure 6b. A small amount of polymer binder was found remaining on the surface of the AP particle from Figure 6c, meanwhile, the damage or fracture of the AP particle can hardly be noticed. From Figure 6d, the extended strip-shape polymer binder implies that severe tearing of the binder matrix occurred under impact loading. It can be concluded that the dominating damage mechanism of H-80W is debonding and matrix tearing at a strain rate of 2460 s^−1^ and room temperature. The reason is that at a strain rate of 2460 s^−1^, micro cracks initiate and propagate in the binder itself, rather than propagate to the stiffer AP particles, resulting in the phenomenon of debonding and matrix tearing. During the test of 2460 s^−1^, no macroscopic fracture occurs because of the input energy is insufficient. For characterizing the complete fracture process of H-80W by SHPB, a higher strain rate is needed (increasing the loading speed). A representative true stress–strain plot up to fracture was acquired under a strain rate of 6100 s^−1^, as depicted in Figure 7.

Figure 7 illustrates the true stress-strain curve of H-80W under a strain rate of 6100 s^−1^, causing a final fracture. Compared with the curve of strain rate 2460 s^−1^, the curve of strain rate 6100 s^−1^ exhibits much higher flow stress, ultimate stress and strain energy density. The propellant exhibits strong nonlinear mechanical behaviors under a strain rate of 6100 s^−1^, without an obvious yielding. The ultimate stress and strain energy density in this test can be identified as 19 MPa and 16.43 MJ/m^3^, respectively, and the strain associated with ultimate stress is 1.0. At the final part of the curve, the stress falls sharply, corresponding to the loss of capacity of loading induced by the final fracture. In addition, compared with the curve of Figure 4, the curve of Figure 7 exhibits an obvious plateau stress region, which means a region with a rapid increase of strain but a moderate rise in stress. As Figure 7 depicted, the plateau stress is almost consistent with 17 MPa at a strain ranging from 0.4 to 0.8. It is well documented that the plateau stress region of particle-filling composites (such as HTPB propellant) can be attributed to a multiple damage mechanism under dynamic loading, including particle dewetting, craze propagation and particle cracking [36].

**Figure 8:**
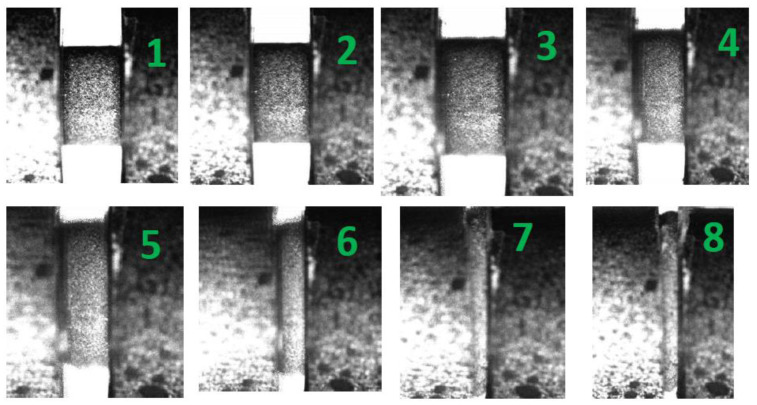
Deformation and fracture processes of H-80W recorded by a high-speed digital camera under the strain rate of 6100 s^−1^, the green numbers in the images correspond to the numbers marked in Figure 7.

**Figure 9:**
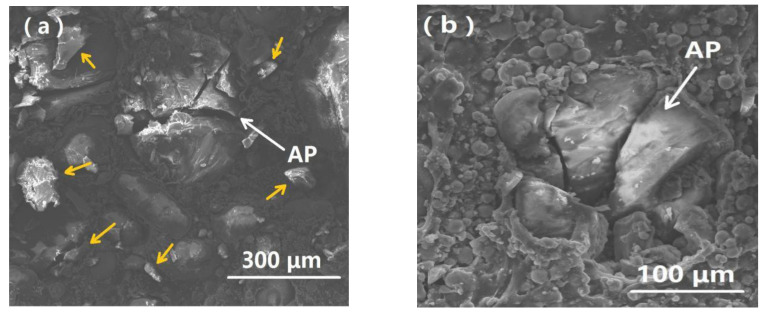

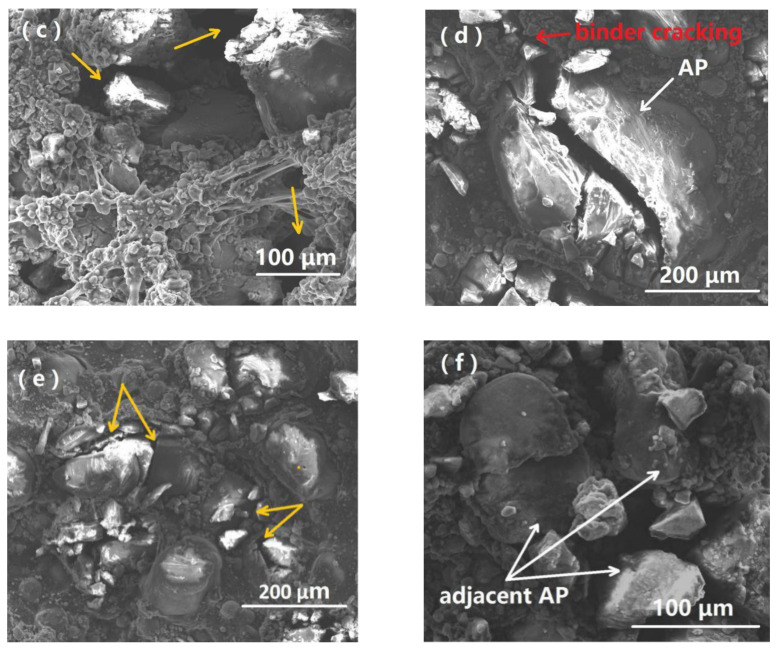
SEM micrographs of the fractured H-80W after SHPB testing at room temperature under a strain rate of 6100 s^−1^: (**a**) cracking of AP particle and pieces of broken AP spreading on the fracture surface; (**b**) individual cracking AP particle without direct contact with other particles; (**c**) the multiple porosity and binder matrix tearing induced by high-speed impact loading; (**d**) cracking propagating through binder matrix into AP particles; (**e**) cracking propagating through the AP particle-binder interface into the AP particles; (**f**) cracking propagating through the adjacent AP particles.

Similar to the study of lower strain rate testing, Figure 7 showed the stress–strain evolution with green spots corresponding to the high-speed images, as shown in Figure 8. A nearly homogeneous uniaxial compressive procedure over the whole loading history can be observed under a strain rate of 6100 s^−1^, which produced a large deformation. As the strain increased, the crack initiated and the fractured debris were squeezed out from the two bars according to images No. 7 and No. 8, which implies the final collapse of the specimen. The post-test SEM was employed to investigate the damage mechanisms at a strain rate of 6100 s^−1^. The SEM recordings are revealed in Figure 9, and the direction of impact loading is perpendicular to the images.

The analysis of microscopic morphology on the fractured sample indicated that the damage of H-80W is more serious at a strain rate of 6100 s^−1^ than that of 2460 s^−1^ because the increase of strain rate can cause a higher loading level and a higher input strain energy. Obviously, the specimen underwent total collapse, which can be proven by the cracking of the AP particle, and the broken AP debris scattering over the vision, as pointed out by yellow arrows in Figure 9a. The fracture surface of AP particles exhibits a characteristic feature of rapid crack propagation, which behaves in a brittle manner according to Figure 9a. In addition, higher applied stress, caused by high strain rate loading, can be transferred from HTPB matrices to the AP particles, even leads to cracking of individual AP particles without direct contact with other particles, because it is easier for cracks to initiate, nucleate and propagate in the AP particles when increasing strain rate, as presented in Figure 9b [43]. The dark regions in Figure 9c represent the porosity of the specimens induced by high-speed impact, forming a lot of voids in the specimen, as pointed by yellow arrows. Meanwhile, the phenomenon of the binder matrix tearing can also be found in Figure 9c. The multiple cracking mechanism of the specimen can be explored by analyzing the modes of crack initiation and propagation. Three typical crack propagating paths can be revealed in detail. Firstly, cracking through the binder matrix into the AP particle, inducing a final transcrystalline damage, as shown by the red arrow in Figure 9d. Secondly, cracking through the AP particle-binder interface into the AP particle, as shown by the yellow arrow in Figure 9e. Thirdly, cracking through the adjacent AP particles (see Figure 9f). In summary, the multiple cracking paths indicate a multiple damage mechanism, which can be due to the larger damage energy of HTPB propellant under higher strain rate. By comparing the complete stress–strain curves and SEM records of HTPB propellant under two representative strain rate loadings, 2460 s^−1^ (without inducing fracture) and 6100 s^−1^ (inducing fracture), a complete damage morphology and mechanical response can be obtained.

### 3.2. Strain-Rate Dependence on the Dynamic Mechanical Response

**Figure 10:**
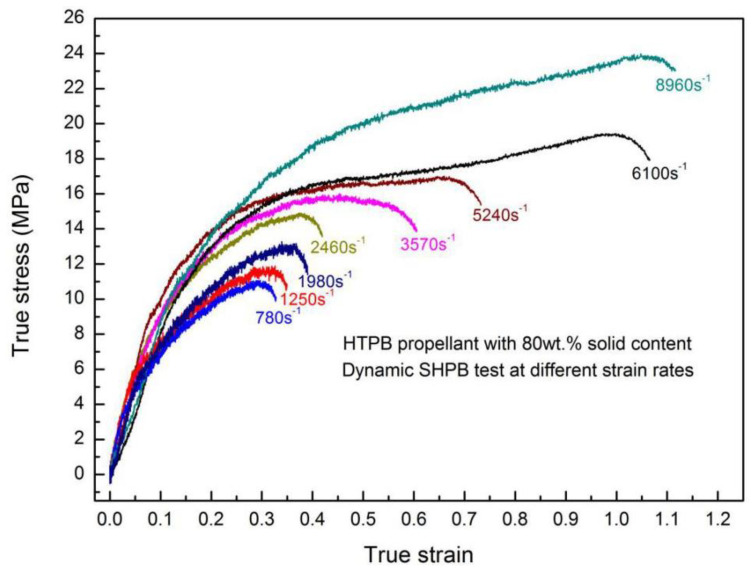
True stress–strain plots of H-80W under various strain rates.

To explore the strain-rate dependency on the mechanical properties of HTPB propellant, various strain rates of dynamic tests were conducted on H-80W by SHPB, as shown in Figure 10. The ultimate stress (from 11 MPa to nearly 24 MPa) and the associated strain (from 0.3 to 1.1) can be determined from these stress–strain plots directly. From Figure 10, as strain rate increases, the final strain, ultimate stress and strain energy density of H-80W increase significantly, which can be attributed to the higher applied stress and larger energy consumption of a specimen under higher rate loading. Meanwhile, the activation energy of a molecular segment movement can be promoted as increasing the strain rate, which leads to a rising of ultimate stress. As shown in Figure 10, the stress–strain curves of H-80W at strain rates ranging from 780 to 8900 s^−1^ exhibit a similar feature, which can be defined as three regions in sequence: initially linear elastic, followed by strain hardening, and finally stress failure. The similar features of the curve indicated that the binder matrix exerts a dominant effect on the dynamic mechanical response of the HTPB propellant.

**Figure 11:**
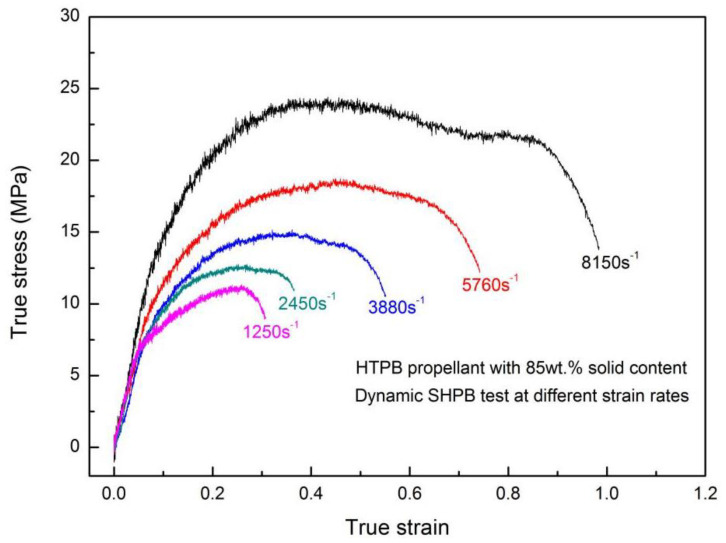
True stress–strain plots of H-85W under various strain rates.

In order to study the effect of solid particle density on the dynamic response of HTPB propellant, another HTPB propellant with particle content of 85 wt.% (H-85W) was subjected to SHPB tests at strain rates ranging from 1250 to 8150 s^−1^, as shown in Figure 11. For the high rate loading using the SHPB technique, the strain rates are mainly controlled by the velocity of a striker bar. It is rather difficult to repeat the strain rates in every test exactly. Note that the deviation of the counterpart strain rate in Figure 10 and Figure 11 is 10% around, for example, 8960 and 8150 s^−1^, which has a limited effect on the stress–strain performance [44]. From Figure 11, there is somewhat of a difference between the stress–strain plot of 8150 s^−1^ and the other plots, which can be attributed to the extensive cracking of AP particles under high-speed loading. By comparing the mechanical properties of H-80W and H-85W, the final strain, ultimate stress and strain energy density of H-80W display greater values than H-85W at strain rates ranging from 1250 to 8150 s^−1^. The main reason is that the weak point in the propellant lies in the interface of the particle-binder, which is more dominant in H-85W because of the larger AP particle density [45].

In order to assess the effect of strain rate on the stress of materials, the strain-rate sensitivity (SRS) index, m, is introduced and can be expressed as below by Equation (3):

(3) $$m=\frac{ln(\sigma /\sigma^{0})}{ln(\dot{\varepsilon }/{\dot{\varepsilon }}^{0})}$$

in which, $\dot{\varepsilon }$ is strain rate; σ is stress, σ^0^ and $\dot{\varepsilon }$_0_ are reference stress and reference strain rate, respectively. Equation (3) can be simplified to Equation (4), a well-known empirical power law function to represent the strain rate dependency on the stress of various materials, which can be defined as the Backofen formula [46,47].

(4) $$\sigma =K\sigma \cdot {\dot{\varepsilon }}^{m}$$

Herein, *K_σ_* is an intrinsic parameter of material, which is related to factors such as strain, temperature and microstructure of material. This power law relation is also available to represent the strain rate dependency on strain energy density, given by Equation (5):

(5) $$U=Ku\cdot {\dot{\varepsilon }}^{m}$$

Herein, *U* is strain energy density; $\dot{\varepsilon }$ is strain rate; *K_u_* is an intrinsic parameter of material related to strain energy density and m is the SRS index.

**Figure 12:**
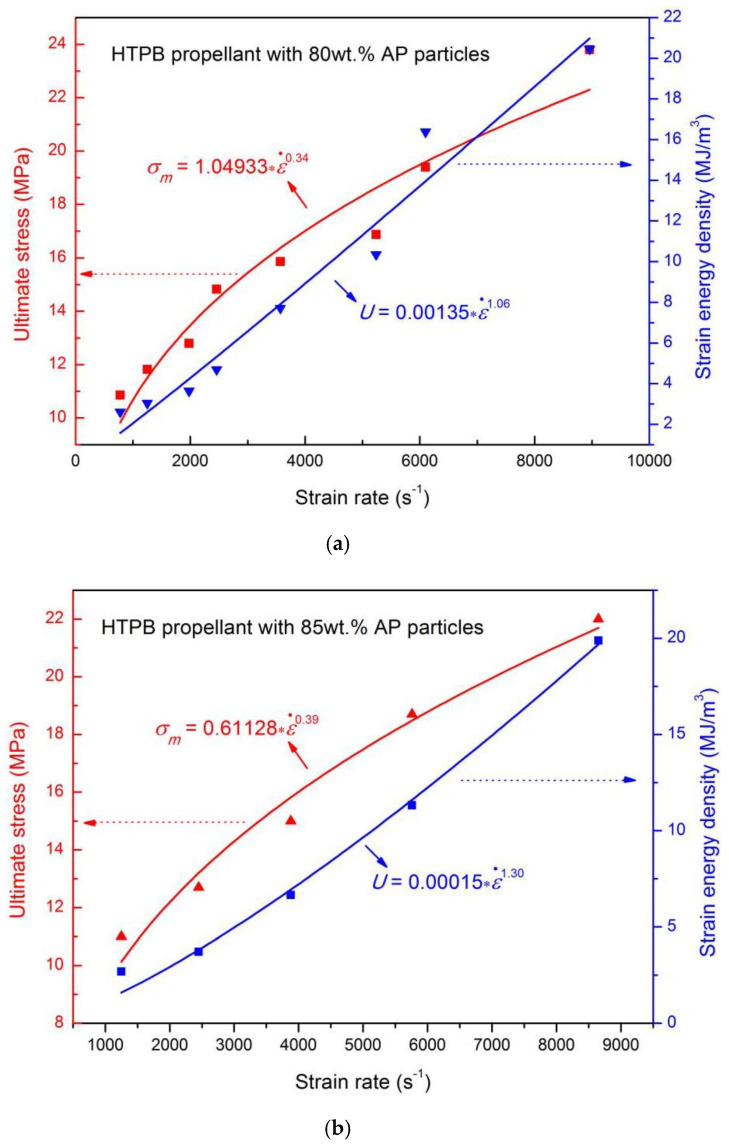
The strain rate dependence on ultimate stress and strain energy density of (**a**) H-80W and (**b**) H-85W.

To quantitatively compare the influence of strain rate on the mechanical properties of H-80W and H-85W, ultimate stress and strain energy density are plotted as a function of strain rate respectively, presented as Figure 12a,b. For the H-80W, the rate-dependent relations of ultimate stress, *σ_m_* and the input of strain energy density, *U* are given by: *σ_m_* = 1.04933$\dot{\varepsilon }$^0.34^ and *U* = 0.00135$\dot{\varepsilon }$^1.06^ by fitting the power law function, respectively (see Figure 12a). For the H-85W, the rate-dependent relations of ultimate stress and the input of strain energy density can be expressed as, *σ_m_* = 0.61128$\dot{\varepsilon }$^0.39^ and *U* = 0.00015$\dot{\varepsilon }$^1.30^ by fitting the power law function, respectively (see Figure 12b). Both fitted curves show an obvious increasing tendency with strain rate, while the amplitude of increase is different. To compare the different strain rate dependence of H-80W and H-85W in more detail, the material parameter, *K*, and SRS index, *m*, have been listed in Table 2.

In the power law relation of Equation (4), the data of *K_σ_* represents the parameter value of HTPB propellant at low strain rate (provided that strain rate $\dot{\varepsilon }$ is 1s^−1^). For the ultimate stress, the parameter, *K_σ_* of H-85W (0.61128) is much lower than that of H-80W (1.04933). It implies the decreased load-bearing capacity of H-85W at a low strain rate level related to H-80W, because a higher content of AP particles is harmful to the load-bearing capacity of the propellant at low strain rate. Whereas, for the parameter, *m* of H-85W (0.39) is higher than that of H-80W (0.34). It indicated a faster increased ultimate stress with increasing strain rate for H-85W, which can be attributed to the greater load-bearing capacity of H-85W than H-80W under high strain-rate loading, because of the skeleton effect of the AP particle. Furthermore, the cracking of AP particles also leads to a more rapid growth in strain energy density with the increase of strain rate, which can be proven by a higher m value for the strain energy density of H-85W (1.30) than that of H-80W (1.06). Based on the study of the rate-dependence of H-80W and H-85W, it can be concluded that, compared with H-80W, H-85W exhibits lower ultimate stress and strain energy density, while the strain-rate sensitivity of ultimate stress and strain energy density are higher for H-85W. The reason can be attributed to the more severe fracture process (including multiple cracking modes of AP particles) for H-85W under high strain-rate loading, because of the higher AP particle density and lower binder content for H-85W than H-80W.

**Figure 13:**
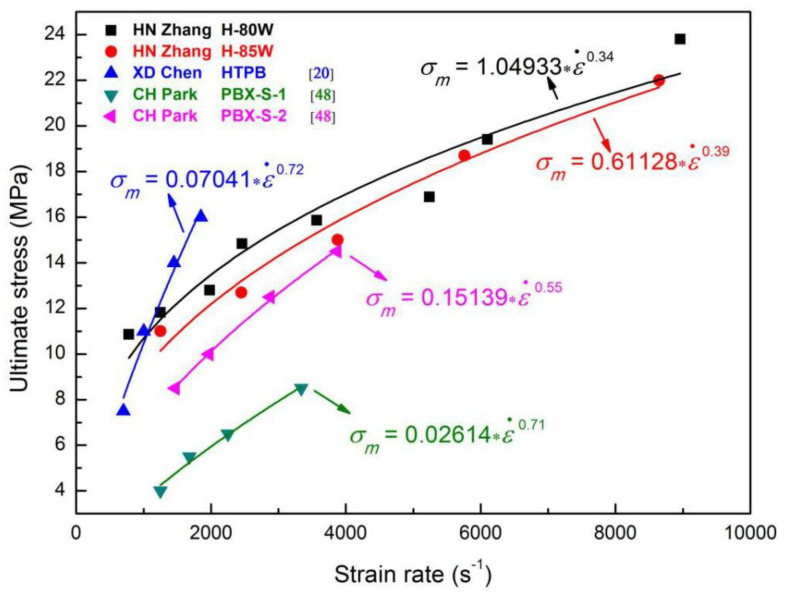
The rate-dependent relations of ultimate stress fitted by Equation (3) for H-80W, H-85W and other reported HTPB based materials.

As presented in Figure 13, the stress–strain data of other reported HTPB based materials were also fitted by Equation (4), to obtain a series of power functions related to the rate-dependence of ultimate stress, which verifies the validity of the simple empirical formula in dynamic loadings [20,48]. As shown in Figure 13, compared with the other works, the experimental strain rate range referred to in this study is much wider, which means that a more complete mechanical response of HTPB propellant can be obtained during high-speed impact loading.

## 4. Conclusions

The dynamic mechanical properties and damage mechanism of HTPB propellant was determined by an SHPB setup synchronizing with a high-speed camera. The detailed conclusions can be drawn as follows.

For the HTPB propellant with embedding 80 wt.% particle content (H-80W), the profiles of the true stress–strain plots with strain rates spanning from 780 to 8960 s^−1^ at room temperature are similar, which indicated an identical deformation process. Final strain, ultimate stress and strain energy density of HTPB propellant all show an evident increasing tendency with strain rate. In addition, SEM was applied to study the microscopic damage mechanisms of the specimen at two representative strain rates, 2460 s^−1^ (no-fracture) and 6100 s^−1^ (fractured). The dominating damage mechanism of HTPB propellant at strain rate of 2460 s^−1^ is debonding and matrix tearing, whereas in the case of 6100 s^−1^, a more serious fracture with multiple cracking modes of AP particles was induced.

Furthermore, the influence of AP particle density on the dynamic response of HTPB propellant was also investigated. The difference of the strain–stress data between H-80W (80 wt.%) and H-85W (85 wt.%) was quantitatively compared, not only based on the ultimate stress and strain energy density, but also the material parameter (*K*) and strain-rate sensitivity index (*m*) fitted by a power law function. In comparison with H-80W, H-85W exhibits a reduced ultimate stress and strain energy density, because of the lower binder content for H-85W. However, H-85W reveals a higher rate-dependence on ultimate stress and strain energy density than H-80W, which can be attributed to the greater load-bearing capacity of H-85W under high strain-rate loading, because of the skeleton effect of the AP particle. In conclusion, the dynamic mechanical response and the multiple damage mechanisms deduced by this study can produce a fundamental scientific interest for developing a new HTPB propellant with good structural integrity and safety.

## Figures and Tables

**Table 1:** Formulation of two Hydroxyl-terminated polybutadiene (HTPB) propellants with 80 wt.% and 85 wt.% filler density.

Formulation	HTPB	AP	Al	DOA	Other Additives
H-80W	12	65	15	4	4
H-85W	9	70	15	3	3

**Table 2:** Parameters of strain rate dependence on ultimate stress and strain energy density described by a power function in Equations (4) and (5) for H-80W and H-85W.

Sample	Ultimate Stress (σ)		Strain Energy Density (*U*)	
	*K* *_σ_*	*m*	*K_u_*	*m*
H-80W	1.04933	0.34	0.00135	1.06
H-85W	0.61128	0.39	0.00015	1.30
